# Topology optimization search of deep convolution neural networks for CT and X-ray image classification

**DOI:** 10.1186/s12880-022-00847-w

**Published:** 2022-07-05

**Authors:** Hassen Louati, Ali Louati, Slim Bechikh, Fatma Masmoudi, Abdulaziz Aldaej, Elham Kariri

**Affiliations:** 1 SMART Lab, University of Tunis, ISG, Tunis, Tunisia; 2 Department of Information Systems, College of Computer Engineering and Sciences, Prince Sattam Bin Abdulaziz University, 11942, Al-Kharj, Saudi Arabia

**Keywords:** DCNN, Optimization, Topologies, Pruning, CT images, XRAY images

## Abstract

Covid-19 is a disease that can lead to pneumonia, respiratory syndrome, septic shock, multiple organ failure, and death. This pandemic is viewed as a critical component of the fight against an enormous threat to the human population. Deep convolutional neural networks have recently proved their ability to perform well in classification and dimension reduction tasks. Selecting hyper-parameters is critical for these networks. This is because the search space expands exponentially in size as the number of layers increases. All existing approaches utilize a pre-trained or designed architecture as an input. None of them takes design and pruning into account throughout the process. In fact, there exists a convolutional topology for any architecture, and each block of a CNN corresponds to an optimization problem with a large search space. However, there are no guidelines for designing a specific architecture for a specific purpose; thus, such design is highly subjective and heavily reliant on data scientists’ knowledge and expertise. Motivated by this observation, we propose a topology optimization method for designing a convolutional neural network capable of classifying radiography images and detecting probable chest anomalies and infections, including COVID-19. Our method has been validated in a number of comparative studies against relevant state-of-the-art architectures.

## Introduction

COVID-19 is an infectious disease caused by severe acute respiratory syndrome [1] and is referred to as the coronavirus due to its appearance. The war on COVID-19 has pushed researchers worldwide to examine, comprehend, and invent novel diagnostic and treatment methods in order to eliminate this generation’s greatest menace. Indeed, the chest X-ray is one of the most commonly used radiological tests for diagnosing a variety of lung diseases. Indeed, numerous X-ray imaging studies are archived and aggregated in many image archiving and communication systems throughout several modern hospitals. An open question arises: how can a database holding priceless image data be used to help the development of data-starved deep learning models for computer-assisted diagnostic systems? There are few published studies devoted to detecting the chest radiograph imaging view [2]. Deep learning has made remarkable strides in a variety of computer vision challenges. During the last decade, deep learning has taken important steps in several domains such as transportation [46, 51], emergency prediction [49, 50], Computer Vision Applications including the classification of natural and medical images [2, 3]. This accomplishment has inspired numerous researchers to use deep convolutional neural networks to diagnose chest diseases in chest radiography (DCNNs) [53]. Despite CNNs’ great performance, their architectural design remains a serious challenge for researchers and practitioners. The CNN architecture is defined by a large number of hyperparameters, which need to be fine-tuned to optimize the design. Several CNN designs have been presented over the last eight years by experienced engineers at well-known organizations like Google. ResNet [4], AlexNet [4] and VGGNet [5] are a few examples. Due to the fact that these structures were created manually, researchers in the fields of optimization [45, 48] and machine learning [47] hypothesized that improved architectures could be discovered using automated methods. In fact, back propagation learning has often been shown to be inefficient in multi-layered networks due to the method being trapped in local minima by gradient descent. Indeed, some researchers [6, 7] have proposed novel learning approaches, most frequently layer per layer, to overcome the practical limits of back-propagation and to maximize the internal representation potential of deep networks. All existing methods employ trained or designed architecture as input. None of them takes into account design and pruning throughout the process. In fact, a convolutional topology exists for every architecture, and each CNN block corresponds to an optimization problem with a large search space [52]. However, there are no guidelines for designing a particular architecture for a particular purpose; as a result, such design is highly subjective and heavily dependent on the knowledge and experience of data scientists. Our goal is to define an automatic method to optimize the hyper-parameters of DCNNs, particularly those controlling their topology. The target is to optimize the number of hidden layers and the number of their associated neurons. Each layer has its own hyper-parameters; hence, the size of the search space increases exponentially with the number of layers. After finding the optimal CNN topologies, we determine the optimal number of neurons for each of the hidden layers before proceeding with the learning for the next layer. This procedure enables the search space’s cardinality to be reduced. Our study finds the appropriate number of neurons per layer after the design of a CNN architecture in order to create a suitable architecture with the least amount of complexity for chest X-ray and CT Image Classification based on COVID-19 Diagnosis. The main contributions of our paper could be summarized as follows:

- Genetic algorithms are used to design CNN architectures that are dependent on the following: (1) hyperparameter settings; and (2) the graph topologies of convolution nodes.
- For the first time, an evolutionary method that combines CNN architecture generation with neural network pruning for resizing a deep learning model by combining the removal of ineffective components.
- Examine the usefulness and adaptability of the generated optimized architecture for X-ray and CT image classification.

## Related work

### Topology optimization for deep neural networks

In recent years, evolutionary optimization for CNN design has been successfully used for many machine learning tasks. According to previous research, this success can be attributed to population-based metaheuristics’ global search capability, which allows them to avoid local optima while finding a near-globally optimal solution. Shinaozaki et al. [24] optimized a DNN’s structure and parameters using GA. While GA works with binary vectors that reflect the structure of a DNN as a directed acyclic graph, CMA-ES, which is fundamentally a continuous optimizer, converts discrete structural variables to real values through an indirect encoding. Xie et al. [25] optimized the recognition accuracy by representing the network topology as a binary string. The primary constraint was the high computing cost, which compelled the authors to conduct the tests on small-scale data sets. Sun et al. [26] proposed an evolutionary method for optimizing the architectures and initializing the weights of convolutional neural networks (CNNs) for image classification applications. This objective was accomplished via the development of a novel weight initialization method, a novel encoding scheme for variable-length chromosomes, a slacked binary tournament selection technique, and an efficient fitness assessment technique. Lu et al. [27] proposed a multi-objective modeling of the architectural search issue by minimizing two potentially competing objectives: classification error rate and computational complexity, quantified by the number of floating-point operations (FLOPS). In Tables 1 and 2, we summarize respectively the X-ray and CT images based COVID-19 as detailed in [3].

**Table 1:** Representative works for X-ray images based COVID-19 diagnosis according to [3]

References	Model of classification	Dataset
Gaal et al. [14]	U-Net + adaptive histogram equalization with adversarial and contrast limits	247 pictures obtained from the Japanese Society of Radiological Technology and 662 chest X-rays obtained from the Shenzhen dataset
Abbas et al. [15]	Decompose, transfer, and compose CNN features of pre-trained models using ImageNet and ResNet + (DeTraC)	80 typical CXR samples
Narin et al. [16]	Transfer learning on a pre-trained ResNet50 model	Dr. Joseph Cohen’s public GitHub repository
Wang et al. [17]	COVID-Net	16,756 chest radiography pictures were collected from 13,645 patients
Hemdanet al. [18]	COVIDX-Net	COVID-19 cases provided by Dr. Adrian Rosebrock
Asnaoui et al. [20]	VGG16, VGG19, DenseNet201, Inception-ResNet-V2, InceptionV3, Resnet50, MobileNet-V2, and Xception have been fine tuned	5856 pictures, 4273 of which are pneumonia and 1583 of which are normal
Sethy et al. [13]	Deepfeatures fromResnet50 and SVM classification	–
Ioannis [23]	Various fine-tune models: VGG19, MobileNet, Inception, Inception Resnet V2, Xception	1427 X-ray images
Ghoshal et al. [22]	Dropweights based Bayesian Convolutional Neural Networks	5941 pictures of PA chest radiography divided into four groups Normal: 1583, Bacterial Pneumonia: 2786, Viral Pneumonia not caused by COVID-19: 1504, and COVID-19: 68
Farooq and Hafeez [21]	To boost model performance, they used a pre-trained ResNet50 architecture with the COVIDx dataset	COVIDx

**Table 2:** Representative works for CT based COVID-19 diagnosis according to [3]

References	Classification model	Segmentation model	Dataset	Number of participants
Song et al. [28]	Details DRE-Net and ResNet50 neural networks for relationship extraction, including Feature Pyramid Network and Attention module	–	777 CT images	COVID-19 infection was identified in 88 individuals (101 infected with bacteria pneumonia, and 86 healthy persons)
Gozes et al. [29]	The design of this 2D Deep convolutional neural network is based on Resnet-50	U-net architecture for image segmentation	–	COVID-19 confirmation of 55 patients
Shan et al. [31]	–	Segmentation of COVID-19 infection areas using a VB-Net neural network	249 CT scans	249 patients were validated using the COVID-19
Jin et al. [32]	–	AI system based on two-dimensional CNNs; the model’s name is not specified	960 computed tomography images	496 patients verified with the COVID-19
Barstugan et.al [33]	Matrix of Grey Level Size Zones SVM + Discrete Wavelet Transform	–	150 CT images	–
Li et al. [34]	COVNet	Segmentation using U-Net	4356 CT images	Six hospitals and 3322 people were included in the databases
Zheng et.al [35]	3COVID-19 Detection Using a Deep Convolutional Neural Network	Segmented with the aid of a pre-trained UNet	–	540 patients
Jin et al. [36]	On ResNet-50, transfer learning is possible	Segmentation model as a three-dimensional U-Net++	–	723 COVID-19 positives

### Deep neural network for COVID 19 control

Over the last decades, CNN for Xray images classification has shown its effectiveness, outperformance, and importance in the field of medical diagnosis. Several computational approaches exist for diagnosing a variety of thoracic diseases using chest X-rays. Wang et al. [8] created a framework for semisupervised multi-label unified classification that incorporates a variety of DCNN multi-label loss and pooling methods. Islam et al. [9] developed a collection of several sophisticated network topolo85 gies to increase classification accuracy. Rajpurkar et al. [10] proved that a standard DenseNet architecture is more accurate than radiologists in detecting pneumonia. Yao et al. [11] developed a method for optimizing the use of statistic label dependencies and thus performance. Irvin et al. [12] developed CheXNet, a deep learning network that makes optimization manageable through dense connections and batch normalization. Prabira et al. [13] collected a set of deep features using nine pre-trained CNN models and then passed them to an SVM (Support Vector Machines) classifier.

## Proposed approach

Our approach is motivated by the following questions:

1. *RQ1* There are an infinitely large number of potential topologies for CNN convolution blocks’ graphs, defining the relationships between nodes. How to determine the best block topology sequence for X-ray images?
2. *RQ2* Any architecture has a huge number of parameters; how can the number of parameters be reduced and the structure architecture reconstructed?

**Fig. 1:**
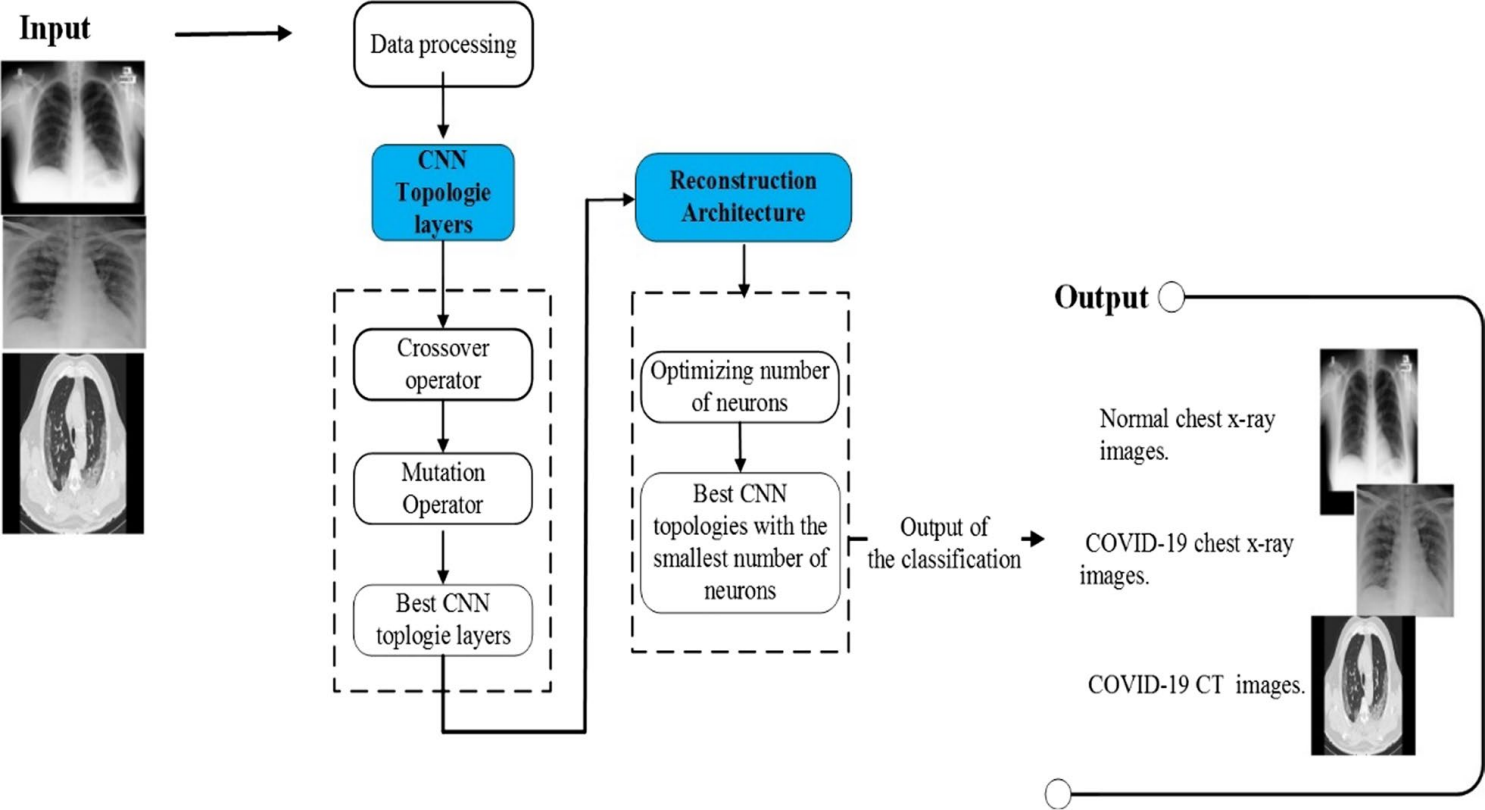
Overview of the proposed CNN for X-ray images classification based EAs

To address these research questions, we must first determine the optimal graph topology sequence for classifying X-ray and CT images and detecting COVID-19 infections, and then reconstruct and discover an optimal number of neurons while maintaining the size of the previous layer. In addition, we are seeking to evaluate the layer’s performance. This requires the ability to compare various topologies. However, such an unusual methodology raises several concerns. Indeed, we can query the existence of a single global optimum for layer size. Depending on the criterion used, there may be multiple or even an infinite number of global optima. With the typical objective of a model selection procedure being to find the most efficient model in terms of performance but also the least computationally intensive, we will seek to establish a lower bound on the number of neurons required for each hidden layer. Indeed, there is no guarantee that the hyper-parameter optimization problem can be separated from layers using the iterative, layer-by-layer method that we propose. In fact, optimizing all hyper-parameters simultaneously in a very deep network would be prohibitively expensive. Therefore, we will investigate the separability of the first two hidden layers. If the result of a global optimization is the same as the hyper-parameters that were found through layer-by-layer optimization, separability may be a good assumption. Figure 1 illustrates an overview of the proposed CNN for X-ray images classification based on evolutionary optimization.

### CNN topologies layers

The solution encoding is a sequence of squared binary matrices, each of which represents a possible directed graph. An element value equal to 1 means that the row node is a predecessor of the column node, while a value of zero means that there is no connection between the two nodes.

*Crossover operator* we use the two-point crossover operator [37] to vary the population because it allows for variation in all chromosome segments. To implement such an operator, each parent solution must be a set of binary strings [37]. Two cutting points are applied to each parent in the two-point crossover process, and then the bits between the cuts are swapped to obtain two offspring solutions.

*Mutation operator* as with the crossover operator, the solution is converted to a binary string using Gray encoding before applying the one-point mutation [37]. The test error is computed using the holdout validation technique [43], which randomly selects 80% of the data records for training and 20% for testing. To deal with this the over-fitting issue, the training data (80%) is divided into 5 folds, and thus fivefold cross-validation is applied during training. The classification performance is averaged over the 5 folds of the training partitions.

### Reconstruction architecture

In response to the problem of hyper-parameter selection in deep convolution neural networks, we have optimized the topology of a deep neural network and, more specifically, the number of neurons in the hidden layers. Our goal is to discover an optimal number of neurons, layer after layer, with the size of the previous layer being fixed. To validate our approach, we optimize the size of the second layer after setting the size of the layer according to the previous optimum. To perform a reconstruction, we first propagate the input to the highest layer using the conditional probabilities of each convolution. Secondly, the configuration of the highest layer is back-propagated with the conditional probabilities.

## Experiments

### Benchmarks and performance metrics

COVID-19 patients’ chest X-rays were obtained from Dr. Joseph Cohen’s opensource GitHub repository https://github.com/ieee8023/covid-chestxray-dataset. This repository contains chest X-ray images of a variety of patients who have been diagnosed with acute respiratory distress syndrome, severe acute respiratory syndrome, COVID-19, or pneumonia. Our experiment is based on a database of chest radiographic images divided into two categories: non-infected patients and COVID-19-infected patients. The dataset was randomly divided into two independent datasets with 80% for training and 20% for testing.

### Performance metrics

Based on the analysis of the related works, the most used performance metrics in image classification using deep neural networks are the Accuracy (*Acc*), *Specificity* and *Sensitivity* [37]. The *Acc* mathematical expression is given by Eq. (1) where *TP* is the number of true positives, TN is the number of true negatives, and *NE* is the total number examples.

1 $$Acc = {{\left( {TP + TN} \right)} \mathord{\left/ {\vphantom {{\left( {TP + TN} \right)} {NE}}} \right. \kern-\nulldelimiterspace} {NE}}$$

The unbalanced class distribution has been addressed using Geometric Mean metrics derived from the binary confusion matrix. Geometric The mean G-mean is the geometric mean of positive and negative true rates. This measure aims to balance the classification performance of majority and minority classes. This metric is insensitive to data imbalance. Equation 2 illustrates the G-mean formula.

2 $$G - mean = \sqrt {TRP.TNR}$$

### Technical details

**Fig. 2:**
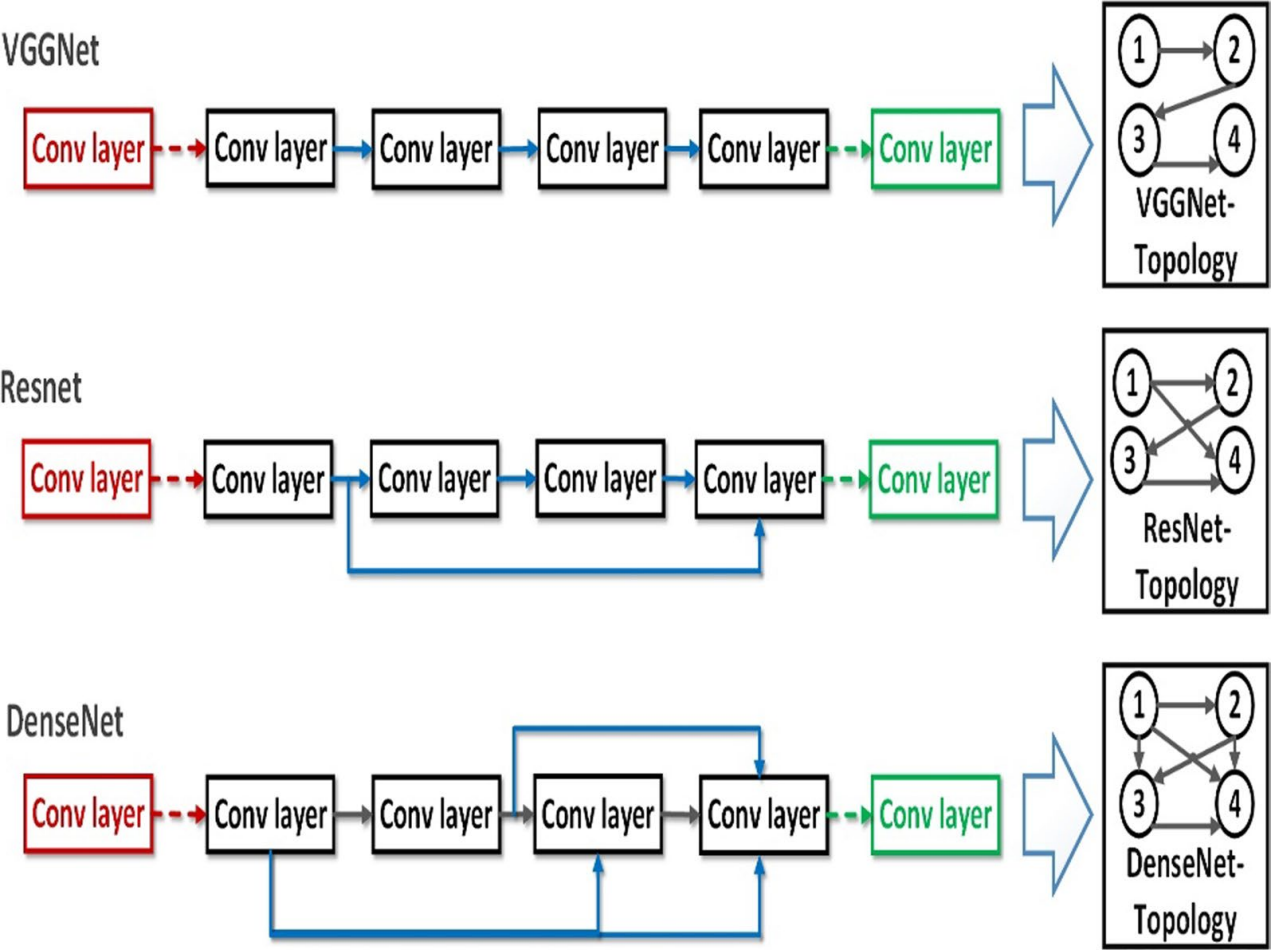
Block topologies of three samples CNNs: VGGNet, ResNet, and DenseNet; for 4 convolution nodes

There exists a topology of convolution within each block of a CNN for any architecture, as illustrated in Fig. 2. This topology corresponds to an optimization problem with a large search space. Numerous CNN architectures already exist, according to the literature. Unfortunately, there are no guidelines for designing a specific architecture for a specific task; as a result, such design remains highly subjective and highly dependent on data scientists’ expertise. As described in Section A, the solution encoding consists of a series of squared binary matrices, each of which represents a possible directed graph. Table 3 summarizes the parameters settings used in our experiments.

**Table 3:** Summary of parameter settings

Categories	Parameters	Value
Gradient descent	Batch size	128
	Epochs	50/350
	SGD learning rate	0.1
	Momentum	0.9
	Weight decay	0.0001
Search strategy	# Of generation	40
	Population size	60
	Crossover probability	0.9
	Mutation probability	0.1

### Optimizing the size of a layer

**Fig. 3:**
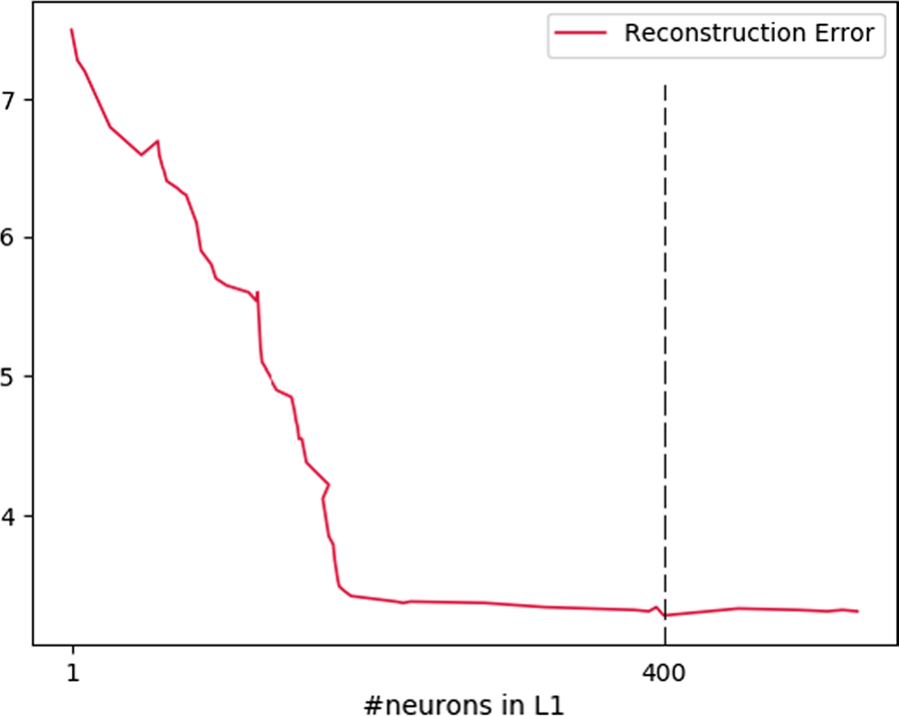
Reconstruction error depending on the size of the hidden layer L1

Figure 3 gives the reconstruction as a function of the number of neurons in the first hidden layer L1 of CNN. The size of the layer, on the abscissa, is presented on a logarithmic scale. It can be seen that the best performance is obtained for the configurations having a minimum of 400 neurons in this hidden layer.

**Fig. 4:**
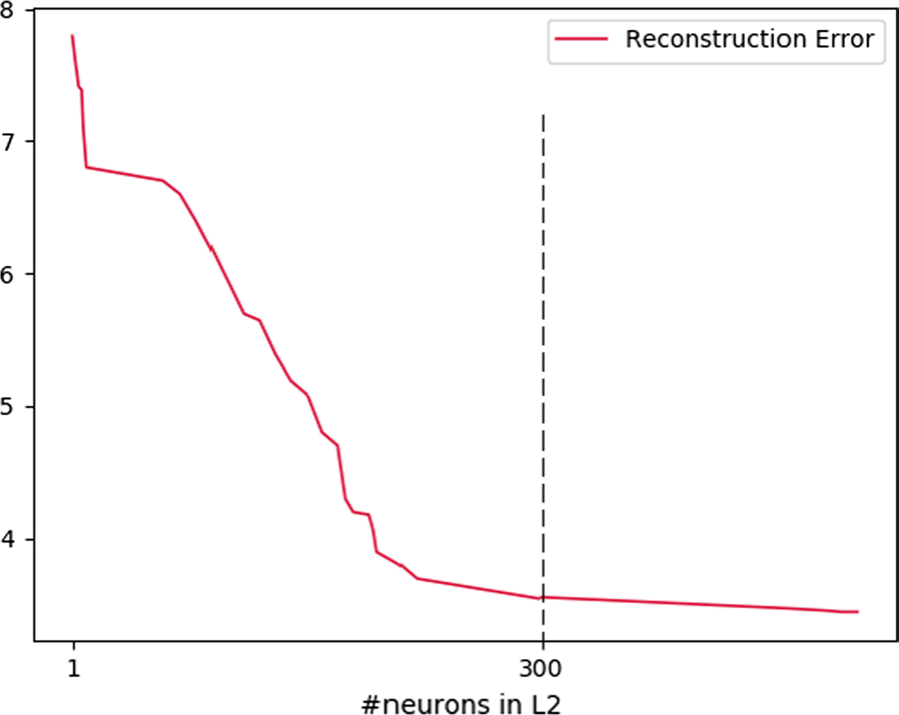
Reconstruction error as a function of the size of the L2 layer, the size of L1 having been set at 400neurons

Moreover, once this minimum is reached, adding more neurons does not significantly increase performance. This observation validates our choice to determine a lower bound for the optimal size of a hidden layer. In fact, to perform a reconstruction, we first propagate the input to the topmost layer using the conditional probabilities. Secondly, the configuration of the highest layer is back-propagated with the conditional probabilities. The reconstruction error is then the distance between the initial entry and the reconstructed entry. To validate the results, we optimize the size of the second L1 layer after setting the size of the L1 layer according to the previous optimum (400 neurons). The lower bounds of the optimal topology, namely 400 neurons on L1 and 300 on L2, are found with the simultaneous optimizations of the two hidden layers, as can be seen in Fig. 4. To validate the results, we optimize the size of the second L1 layer after setting the size of the L1 layer according to the previous optimum (400 neurons). The lower bounds of the optimal topology, namely 400 neurons on L1 and 300 on L2, are found with the simultaneous optimization of the two hidden layers, as can be seen in Figs. 3 and 4.

### Comparative results

Recently, many computational intelligence methods have been proposed for COVID19 detection using X-ray images and Computed Tomography (CT) ones. Our approach is compared to the most representative works of CNN architecture generation methods. Tables 4 and 5 summarizes the obtained comparative results of the different architectures outputted by the confronted CNN design methods on X-ray images.

**Table 4:** Representative works for CT based COVID-19 Diagnosis according to [3]

Study	References	Test Acc (%)	Sensitivity	Specificity	G-mean
Chen et al.	[32]	95.24	100	93.55	92.30
Wang et al.	[8]	82.9	84	80.5	87.45
Xu et al.	[19]	86.7	–	–	88.91
Song et al.	[28]	93	–	–	90.66
Gozes et al.	[29]	94.22	98.2	92.2	92.30
Shan et al.	[31]	91.6	–	–	89.95
Jin et al	[32]	94.98	–	–	92.77
Li et al.	[34]	88.17	90	96	89.45
Jin et al.	[36]	93.58	97	92	92.97
Louati et al.	Our work	96.87	97.58	95.14	96.10

**Table 5:** Representative work for X-ray based COVID-19 diagnosis [3]

Study	References	Test Acc (%)	Sensitivity	Specificity	G-mean
Gaal et al.	[14]	97.5	–	–	97.14
Abbas et al.	[15]	95.12	97.91	91.87	94.69
Narin et al.	[16]	97	–	–	96.78
Wang et al.	[17]	92.4	–	–	91.06
Asnaoui et al.	[20]	96	–	–	95.98
Sethy et al.	[13]	95.38	–	–	94.14
Ioannis et al.	[23]	95.57	0.08	99.99	93.44
Ghoshal et al.	[22]	88.39	–	–	89.91
Farooq and Hafeez	[21]	96.23	–	–	95.81
Louati et al.	Our work	98.12	98.44	96.63	97.90

In fact, Tables 4 and 5 summarize the obtained Acc results on CT images and X-ray ones, respectively. We observe that the Acc of CT based COVID-19 Diagnosis is lying between 82.9 and 99.6%. Shuai Wang et al. [8] corresponds to the worst method and provides an Acc 82.9% with specificity of 80.5% and sensitivity of 84%. Always in terms of classification Acc, Xu et al. [19] provides 86.7%, Song et al. [28] provides 93%, Shan [31] provide 91.6% and Jin et al. [32] provides 94.98%. Always based on Table 4, Chen et al. [32] provides 95.24% with sensitivity of 100% and specificity of 93.55%. We observe that Our work is able to achives better ACC values than the considered peer methods. Furthermore, Table 5 show that the Acc of X-ray based COVID-19 Diagnosis is lying between 88.39% and 97.5%. Ghoshal et al. [22] corresponds to the worst method and provides an Acc 88.39%. Always in terms of classification Acc, Wang et al. [17] provides 92.4% on COVIDx dataset, Abbas et al. [15] provides 95.12% with a sensitivity of 97.91%, a specificity of 91.87%, Sethy et al. [13] provides 95.38% and Apostolopoulos et al. [23] provides 95.57% with sensitivity of 0.08% and specificity of 99.99%. Always in Table 5, Asnaoui et al. [20] show highly satisfactory performance with accuracy 96%, Farooq and Hafeez [21] and Gaal et al. [14] provides 96.23% and 97.5% respectively. We observe that our work is able to achieve better ACC values than the considered peer methods. The following arguments could explain these findings: manually designing CNNs is a time-consuming and complex operation that demands a high level of competence on the part of the user. Even with a high level of expertise, developing a good architecture is not easy due to the vast variety of alternative architectures. Evolutionary approaches outperformed other methods in this and earlier studies because reinforcement learning based approaches have greedy behavior that optimizes the ACC throughout the search process. However, evolutionary approaches are capable of escaping local optima and covering the whole search space because of their global search capability and the probability acceptance of inefficient structures via the mating selection operator. These findings validate our proposed algorithm’s ability to construct task-dependent designs 220 automatically. Indeed, we note that our approach is capable of automatically designing a CNN architecture with higher accuracy values than the peer techniques reviewed.

This might be explained by the fact that designing CNNs is very difficult, even with a high degree of knowledge. On radiographic pictures, automated design approaches outperform manually created systems. The reason for this is because there are an infinite number of alternative architectures. To summarize, the optimization of the network topology has a significant influence on classification performance since each topology determines the interactions between the neural network nodes.

## Diagnosis using X-ray 14 images

### Description and motivation

**Fig. 5:**
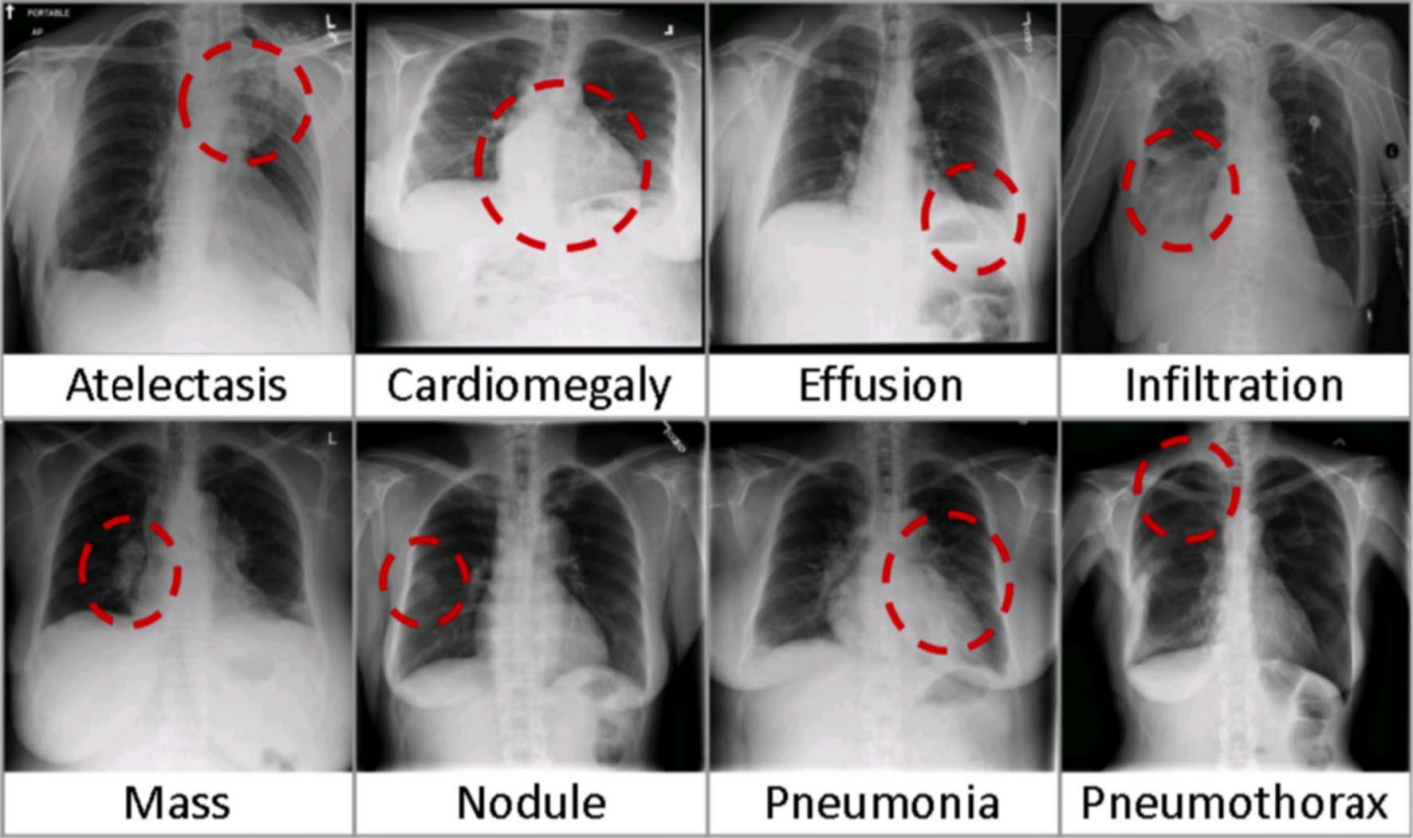
Common thoracic diseases observed in Chest X-ray14 [30]

Chest X-ray14 database consisting of 112,120 frontal-view radiographs X-ray images from 30,805 unique patients. The database was compiled using natural language processing techniques from associated radiological reports stored in hospital image archiving and communication systems. Each image may have one or more common chest conditions (one or many common thoracic diseases), or”Normal” otherwise (see Fig. 5).The dataset is publicly available from NIH at https://nihcc.app.box.com/v/ChestXray-NIHCC.

### Experimentation

**Fig. 6:**
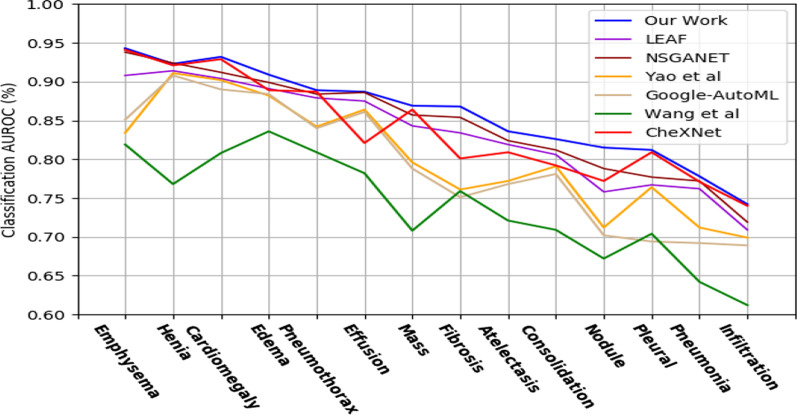
Multi-label classification performance on Chest X-ray14, the class-wise mean test AUROC comparison with peer works

The proposed method is compared to the most representative works in each of the three categories of methodologies for creating CNN architectures (see Fig. 6). The parameters employed in our trials are summarized in Table 6.

**Table 6:** Obtained *AUROC and #Params,* results on Chest X-ray14

Method	Search method	Test AUROC (%)	#Params
Yao et al.	Manual	79.8	–
Wang et al.	Manual	73.8	–
CheXNet	Manual	84.4	7.0 M
Google AutoML	RL	79.7	–
LEAF	EA	84.3	–
NSGANet-X	EA	84.6	2.2 M
Our work	EA	84.91	1.6 M

**Fig. 7:**
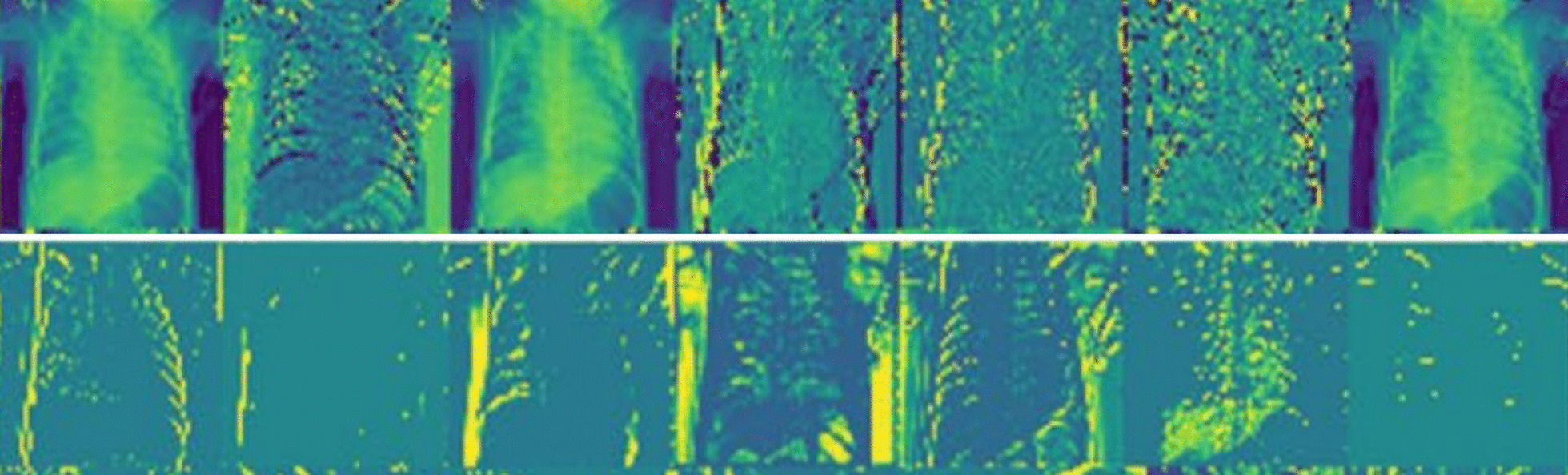
Random sampling of activations is shown in filters of the first and second convolutional layers

Table 6 summarizes the comparative findings achieved for the various architectures developed by the various CNN design approaches when applied to X-ray images. For manual approaches, the AUROC ranges from 79.8 to 84.6%. Google AutoML has the lowest AUROC of any non-manual method, at 79.7 percent.The evolving AUROC curves provide AUROC values of 84.3% for LEAF (2019) and 84.6% for NSGANet-X showing the disease curve of CNN-XRAY and the comparison AUROC by a disease with other peer methods are provided in Fig. 6. We observe that our work is able to automatically design a CNN architecture that achieves better AUROC values than the considered peer methods. Figure 7 illustrates a random sampling of activations shown in filters of the first and second convolutional layers.

## Conclusion

Deep neural networks have demonstrated outstanding performance in a wide range of machine learning tasks, including classification and clustering [39, 40], for real-life applications of soft computing techniques in different fields [41, 42]. Developing an appropriate architecture for a Deep Convolutional Neural Network (DCNN) has remained an extremely intriguing, demanding, and topical issue to date. Following the manual design, many other methodologies have been presented, most of which are based on reinforcement learning and evolutionary optimization, with some adopting a multi-objective perspective. Indeed, there are a huge number of conceivable possible designs with various network topologies. However, since there are no recommendations for designing a specific architecture for a certain task, such design remains highly subjective and heavily dependent on the data scientist’s knowledge. By searching for the ideal sequence of block topologies and reconstructing and determining the optimal number of neurons, layer by layer, to detect COVID-19 infections, we propose an efficient evolutionary technique for designing the CNN architecture in this study. Experiments have shown the efficacy of our proposed technique, which outperforms various typical designs on a data set of CT and X-ray image benchmarks. It is worth emphasizing that the genetic algorithm is computationally expensive, because we need to conduct a complete network training process for each generated individual. Therefore, we run the genetic process on datasets, and demonstrate its ability to find high-quality network structures. It is interesting to see that the generated structures, most of which have been less studied before, often perform better than the standard manually designed ones. Therefore, we need transfer the learned structures such as [44] to large-scale experiments and verify their effectiveness. The approach of TL to overcome the issues of transfer learning from pretrained models of the ImageNet dataset to medical imaging tasks and the annotation process of medical images. Moreover, it will help to address the issue of the lack of training in medical imaging tasks.

## Data Availability

COVID-19 patients’ chest X-rays were obtained from Dr. Joseph Cohen’s opensource GitHub repository https://github.com/ieee8023/covid-chestxray-dataset. This repository contains chest X-ray images of a variety of patients who have been diagnosed with acute respiratory distress syndrome, severe acute respiratory syndrome, COVID-19, or pneumonia. Chest X-ray14 database consisting of 112,120 frontal-view radiographs X-ray images from 30,805 unique patients. The database was compiled using natural 295 language processing techniques from associated radiological reports stored in hospital image archiving and communication systems. Each image may have one or more common chest conditions (one or many common thoracic diseases), or “Normal” otherwise. The dataset is publicly available from NIH at https://nihcc.app.box.com/v/ChestXray-NIHCC. Any additional data could be requested from the corresponding author, Ali Louati.
